# An innovative optics lab design for residency training in ophthalmology

**DOI:** 10.1038/s41598-023-44688-1

**Published:** 2023-12-09

**Authors:** Fateme Alipour, Katayoon Samavati, Parisa Abdi, Mandana Shirazi, Mohammad Taghi Tavassoly

**Affiliations:** 1 Eye Research Center, Farabi Eye Hospital, Tehran University of Medical Sciences, Qazvin Square, Tehran, 1336616351 Iran; 2 Department of Physics, North Tehran Branch, Islamic Azad University, Tehran, Iran; 3 Department of Medical Education, School of Medicine, Tehran University of Medical Sciences, Tehran, Iran; 4 Department of Physics, College of Science, University of Tehran, Tehran, 14395-547 Iran

**Keywords:** Medical research, Translational research

## Abstract

Proper understanding of the optical function of the eye is the foundation of clinical understanding of ophthalmologists. Although teaching principals of optics has always been a part of ophthalmology residency curriculum, it seems that successful strategies other than lecture—based methods are needed to engage students and facilitate the understanding of optical principals. A collaborative team of physicists (optics Ph.D.), ophthalmologists and medical education experts designed an optics lab for ophthalmology residents to help them practically simulate different optical phenomena. The educational course consisted of 4 sessions of 90 min to practice the optical tests using the lab instruments. Each class consisted of 6–9 residents, divided into 3 groups with a fully equipped unit, and two mentors (an optics Ph.D. professor and an ophthalmology professor). A quasi-experimental design with pre-post test was used to evaluate the effectiveness of the training workshop in changing the ophthalmology residents’ optical knowledge and attitude. Thirty-five residents participated in the study. The average score of residents' performance before the workshop was 5.21 (out of 100), which increased significantly to 66.1 after the workshop. Also, the average knowledge of residents, which was measured as self-reported, increased significantly from 28.85 to 71.09. The average score of students' attitudes and interest was increased from 40.49 to 74.81. It seems that training workshops and labs are effective to bring about change in knowledge and attitude of ophthalmology residents toward optics as a new teaching strategy that would be implemented in their curriculum.

## Introduction

Ophthalmology is a specialty aiming better vision as its major goal. To achieve this goal, proper understanding of the optical function of the eye is necessary. Eye is an optical instrument with unique characteristics. So teaching principals of optics has always been a part of ophthalmology residency curriculum. However, considering limited available interventions such as glasses prescription and media opacity removal, and the different background needed for teaching other aspects of optics (physics/optics), other parts of the optics were only briefly and theoretically taught. There are different parts of optics that have many applications in the clinical ophthalmology, such as different types of lasers, the structure and applications of various IOLs, the results of refractive surgeries, etc. However ophthalmologists are usually not familiar with these topics in depth. In recent years, newer and more diverse methods of teaching have become common in the education field including interactive methods, flipped classes, high tech approaches (using AI, 3D printing and virtual reality), but unfortunately, based on the searchable evidences, these methods have not been used much in teaching optics- which is a challenging course in ophthalmology curriculum With advances in diagnostic methods (OCT, aberrometry, topography, …), and medical and surgical interventions (e.g. wave front guided laser ablation, customized intra-ocular lenses, glasses and contact lenses, different available coatings for glasses), this theoretical background appears insufficient.

In order to address this need, in 2010 a decision was made to set up an optics lab for ophthalmology residents and design set ups which help them practically simulate different optical phenomena. In a collaborative (growing) team consisting of physicists (optics Ph.D.), ophthalmologists and medical education experts, a lab course curriculum was written focusing on important aspects of optics for ophthalmologists such as light properties, lenses and prisms, optical aberrations, polarizations and holography to help residents gain a deeper understanding of optical instruments, visual complaints of patients and the impact of their interventions (e.g.: placing an IOL slightly decentered, prescribing eyeglasses with wrong pupillary distance, effect of damaging iris sphincter on depth of field and depth of focus).

Then, a suitable place which we call “optics lab" was equipped with the support of Iranian Society of Physics in Farabi Eye Hospital affiliated to Tehran University of Medical Sciences (TUMS).

For this course, we scheduled 4 sessions for each group of residents (6–9 students) to follow the agenda in three different subgroups (at most 3 residents) with a complete set up, under supervision of optics and ophthalmology professors. Each session started with a quick review of the optical principles which are supposed to be practically simulated in that session by a physic- optics professor. Then, residents practice the designed optics tests, and after each optics test, potential clinical applications of it were discussed by the ophthalmology professor.

Besides the very open and friendly environment which encourages participants to give their feedback at the end of each course, a formal summative written and practical test for evaluating, knowledge, attitude and ability was performed.

These training courses entered the residents' curriculum in Farabi eye hospital in 2011 and have been held as annual workshops for residents. In 2015, it was decided to evaluate the effectiveness of these workshops, so 3 questionnaires were designed to evaluate the change in attitude and performance of residents. Although the optics-lab started working some years ago, this study was conducted from 2017 to 2019.

To the best of our knowledge, this is the first time such a laboratory has been designed to teach optics to ophthalmology residents. Of course, optics laboratories have been designed for other disciplines, including physics students, but there is no record of this in ophthalmology curricula emphasizing their practicality for ophthalmologists.

## Materials and methods

### Research design

A quasi-experimental design with pre-posttest was used to evaluate the effectiveness of the training workshop in changing the ophthalmology residents’ optical knowledge and attitude. This study adhered to the tenets of the Declaration of Helsinki. All participants were informed about the study goals and informed consent was obtained.

### Research setting

The study was conducted from November 2017 to November 2019 in a designed optics lab, Farabi eye hospital, affiliated to Tehran University of Medical Sciences. Two groups of residents in the second and the third year of ophthalmology residency participated in the optics course and in this study as a component of their educational programs.

### Course

This educational course was held in 4 sessions and each session was presented as three separate 10 min lectures given before start of a related series of practices based on a written curriculum. A PDF version of this curriculum was available for all residents before the sessions and a hard copy was given to each resident in the lab.

Lectures reviewed the related optical principles and their applications in practical ophthalmology to attract the attention of participants to key points. Approximately 90 min was considered for practicing the optics tests using the lab instruments. There were 6–9 residents in each class, divided into 3 groups with a fully equipped unit, and a mentor (PhD of optics). An ophthalmology professor in charge of supervising the optics and refraction course in their residency program was present in all sessions taking the responsibility of discussing the relevance of these tests to clinical ophthalmology.

### The lab curriculum

The curriculum was developed by a committee of three (two optics scientists and one ophthalmologist who was in charge of optics and refraction course in the residency program of Farabi Eye Hospital), mainly based on the American Academy of Ophthalmology Clinical Optics book^1^, which is the main reference for the residents. This curriculum took advantage of the experience of the optics professors in designing a proper set up for appropriate optics tests showing the main concepts of optics principles discussed in the above mentioned reference book.

**Figure 1:**
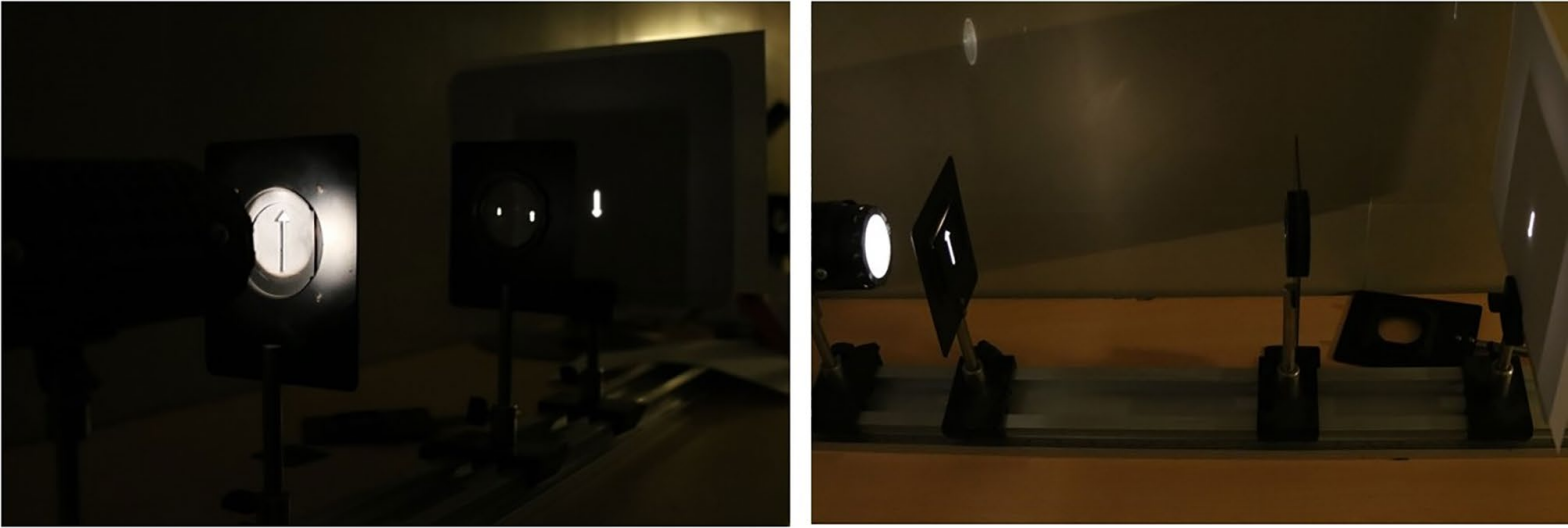
Image formation by convex lens: The image of an arrow is formed by a positive lens on the observation screen. By changing the distance of the object, residents can see the place of image formation and the magnification changes. Also, by moving the lens vertically, the image moves and the prismatic effect of the lens can be observed. In addition, with this experiment, depth of field and depth of focus can be measured.

**Figure 2:**
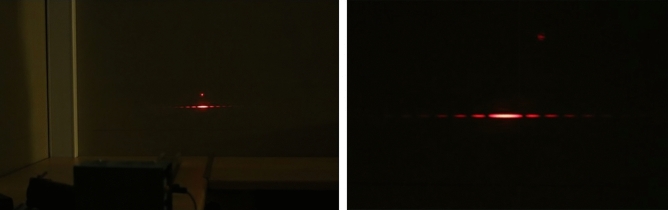
Single-slit diffraction pattern. In this experiment, the Wave nature of light can be shown. As the width of the slit producing a single-slit diffraction pattern is reduced, the diffraction pattern becomes wider and vice versa.

**Figure 3:**
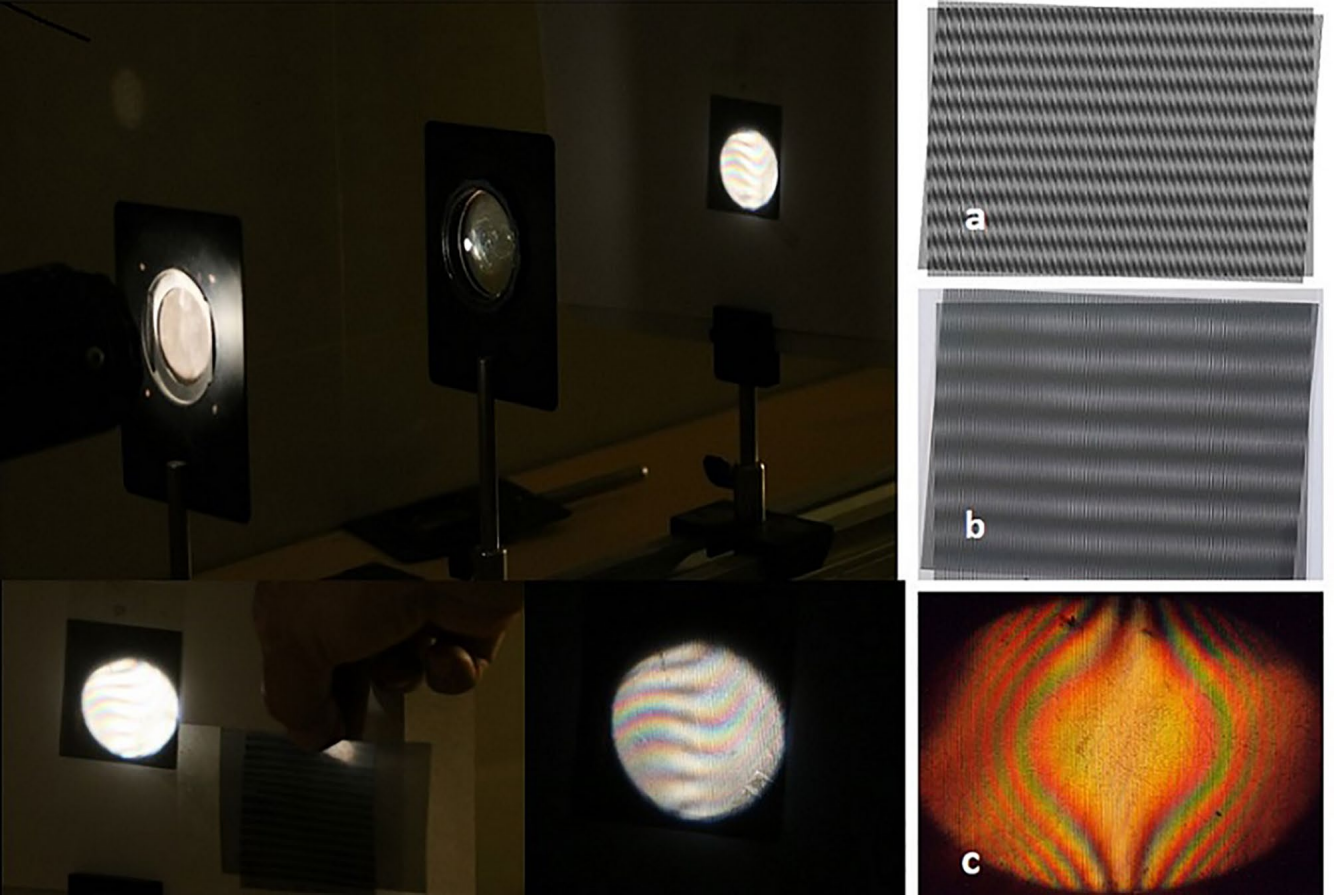
Observation of lens aberration by Moiré technique. The simplest moiré pattern appears when two linear amplitude gratings are superimposed that have equal or nearly equal constant pitches. The pattern is the periodic distribution of reflectance or transmittance of period much larger than the gratings’ pitches. The gratings could be two physical gratings or one physical grating and the image of another grating. Lenses can be tested by using the moiré technique. Using a lens, we form the image of grating in the case that, the distance between the object and screen is 4f. In this case, the grating and the image of grating are similar. By overlaying a grating similar to the first grating over the image of grating, at a small angle, we expect to see linear moiré fringes (**a**). But because of lens aberrations, moiré fringes are not linear. All aberrations, as well as colored aberrations, are seen simultaneously in the moiré pattern (**b**). If the angle is zero, we expect no moiré fringes to form, but because of the aberrations, moiré fringes are created as shown in (**c**). This state is the phase singularity state.

All the practical topics and exercises in the laboratory were designed in 4 main sections: Ray Optics, Optical aberrations, Wave Optics, and Electromagnetic Optics. For each section, the concepts needed by the residents were identified, then appropriate tests were designed and the application of each in the clinical work of an ophthalmologist was determined. Tables 1, 2, 3 and 4 show in detail the concepts of each section, the tests performed for them and the applications of each (Tables 1, 2, 3, 4); also, Figs. 1, 2 and 3 show some of the students’ experiences in the optics lab (Figs. 1, 2, 3).

**Table 1:** Ray optics.

Concepts	Test	Examples of clinical applications
*Law of reflection and refraction of light*		
Refractive index	To investigate Snell's law, we use a transparent semicircular optical flat. The device is adjusted so that light passes through the optical axis and strike the flat side of the semicircular optical flat. The incidence angle and refractive angle are measured and the refractive index is calculated by Snell's law	Understanding ocular media
Law of reflection		Image formation by reflective surfaces like mirrors
Snell’s law		Image formation by lenses
Critical angle and total internal reflection (TI media opacity R)	For showing critical angle the light should strike the curved side of the semicircular optical flat to pass from a media with a higher refractive index to a media with a lower index	Basis of Gonioscopy with three mirror technique
Color dispersion	A prism is used to make a rainbow from a beam of white light	Basis of Duchrome test
Refraction by a prism, Combinations of prisms and ray tracing	Deflection of light through a prism is observed. The deviation angle changes with the rotation of the prism. The minimum angle of deviation is found Ray tracing in a combination of prisms is shown	Understanding image formation by an optical system including prism
Minimum deviation		Determining prisms’ power
Thin prism and power of prism	The laser beam incident on a thin prism is tested and the light deflection is measured at a distance of one meter	Clinical application of prisms
Optical axis of lenses (concave and convex)	Different kinds of thick lenses are put in the way of a ray and focal point on the optical axis is found Then one of the lenses is decentered in different directions to show image displacement	Prismatic effect of decentered lenses e.g., spectacle with wrong PD or decentered intraocular lens
*Thin lenses*		
Image formation	With a concave lens, the image on the viewing screen is formed. For several different positions of the lens the object distance is measured and consequently the image distance and size of image are evaluated. The data are used to check the lens equation. Linear magnification is also calculated from the data. Residents test a number of concave and convex lenses and calculate focal points and focal lengths and lens’ powers	Clinical application in refractive errors
Paraxial approximating		
Use of thin lens formula for converging and diverging lenses		
Lateral magnification		
Focal points and focal lengths and Power of lenses		
Combination of lenses	To obtain the focal length of a negative lens, it is combined with a positive lens. Residents can experience real and virtual image formation	
Pin hole	The viewing screen is adjusted to obtain the best focus of the image. Viewing screen is moved so that the image is still clear. This distance is the depth of focus. In another experiment the object is moved so that the image is still clear. This distance is the depth of field	Understanding the role of senile miosis and some optical instruments such as Kamra inlay or presbyopic IOLs in pseudo -accommodation
Depth of field and depth of focus	When the image is formed by a lens, an aperture is put on that lens, and its effect on the depth of field and depth of focus is investigated Lenses with different power are tested	Clinical application in microscope adjustment

**Table 2:** Optical aberrations.

Concepts	Test	Examples of clinical applications
*Lens aberrations*		
Moiré method for representing aberration	The moiré fringes of the superimposed linear gratings are linear. When a grating is imaged by a lens, due to the aberrations of the lens, the image of grating is not exactly equal to the object, therefore, overlapping gratings over the image does not produce linear moiré	Understanding aberration and its effect on quality of image
Spherical aberration	The viewing screen is adjusted to obtain the best focus of the image. A translucent ring is placed on the lens so that light passes only through the center of the lens. By moving the viewing screen, the best image is found in this mode. The lens power was measured. The experiment is repeated when a circular obstacle is placed on the center of the lens to let only peripheral rays pass. The lens power is measured again. The difference in image distance in these two cases is due to the lateral spherical aberration	Observing different kinds of aberrations and their role on quality of image, such as night myopia, glare, halo, pincushion and barrel distortion
Coma aberration	When a perfect image is formed by a lens, the lens is rotated and tilted. Deviations in the image are due to the coma and astigmatism aberration	
Astigmatism aberration		
Field curvature aberration	The image of a two-dimensional grid is formed by a lens, it is shown that all parts of image could not be focused simultaneously. This is due to Field curvature aberration and Distortion aberration	
Distortion aberration		
Chromatic aberration	When the image is formed by a lens, a color filter is placed into the beam path. It can be seen that image distance is different for different colors. The resident can calculate lateral chromatic aberration by this experiment

**Table 3:** Wave optics.

Concepts	Test	Examples of clinical applications
*Interference of light*		
Huygen’s principle	Huygen’s principle is expressed and we use the wave nature of light to explain some optical phenomena like diffraction, and interference	Anti-reflection lens coating, basis of OCT
Coherent sources	Residents use the laser light in the experiment to see the difference between coherent and non-coherent light	
Spatial coherency	Young’s double-slit experiment is performed	
Temporal coherency	Interference test of reflected rays from the top and bottom plane surface of an optical dielectric film is performed	
*Diffraction*		
Fraunhofer diffraction	Diffraction experiments are performed through two slits, a single slide and a circular aperture	Airy disk & point spread function (PSF)
Fresnel diffraction		
*Holography*		
Hologram of extended object	Residents expand the laser beam by a beam expander. Then, for the purpose of reconstructing the transmission hologram, the residents illuminate the hologram by He–Ne laser, and the three-dimensional image recorded in the hologram is revealed. They reconstruct two transmission holograms by laser light and then a reflection hologram is seen in a white light	Utilizing the unique properties of holography in ophthalmic research
White-light holograms

**Table 4:** Electromagnetic optics.

Concepts	Test	Examples of clinical applications
*Electromagnetic optic concepts and the polarization of light*		
Difference between polarized and unpolarized light	Residents look at the lamplight from behind the polarized sheet and rotate it, no change in light intensity is observed. Then this test is repeated with the reflected light from the floor, in this case, the change in light intensity by rotating the polarized film is obvious Different methods of light polarization, such as: polarization by dichroic polarizer, polarization by reflection from dielectric surfaces, polarization by birefringence materials were tested. A polariscope is used to test isotropic and anisotropic objects and glasses Polarization by reflection is tested using the Nuremberg polarization device	Polarized sunglasses
types of polarization		
Production of polarized light

### Tools of the study

Data were collected using a 3-part self-administered questionnaire, after proving its validity and reliability.

The first part included residents’ demographic characteristics, such as age, gender, course and grade point average (GPA).

A 34-item questionnaire was developed to assess students’ knowledge and attitudes before and after the training workshop. The questionnaire consisted of 2 parts: (1) Knowledge of the residents before and after the workshop. (2) Residents’ attitude towards gaining practical knowledge in the field of optics before and after the workshop.

The knowledge part of the questionnaire concerned residents' prior knowledge of the subject and consisted of two sections: 14 questions on a 5 point Likert scale to assess self-estimation of the residents from their optical knowledge that were scored from 1 to 5; and 6 open-style questions to objectively evaluate the level of their knowledge and performance that were scored from 0 to 24 based on a pre- defined scoring system. The overall knowledge score was classified as 3 levels of knowledge: low, moderate and high.

### Validity and reliability of the questionnaire

The validity of the questionnaire was assessed using panel of experts’ method. For this purpose, the initial questionnaire was designed based on the educational objectives of the workshop and American Academy of Ophthalmology Clinical Optics book using the nominal group technique. Then 3 ophthalmologists read the questionnaire and checked it for comprehensibility (expert opinion for face validity); and 5 experts (2 ophthalmologists, 2 optics specialists and a medical education specialist) assessed and revised the questionnaire for content and compliance with the objectives of the study (expert opinion for content validity).

The reliability of the questionnaire was proved using the test–retest method; 10 residents filled the questionnaire twice with a 2-week interval and correlation coefficient between them (Cronbach's alpha) was 0.85, indicating reliability.

### Study design

Students were asked to complete the questionnaire before the course and one month after the last session. Finally, they were asked to evaluate the quality of the course by a 15-item questionnaire, rating it through a five- point Likert scale ranging from very poor to very strong and to offer their viewpoints and suggestions for further improvement of the course in the future.

### Statistical analysis

Data were represented as descriptive statistics (mean, standard deviation, and frequency table) by SPSS software version 22. The significance level was set at *p* < 0.05.

### Ethics statement

This study has been evaluated in the research ethics committee of Farabi Eye Hospital Tehran university of medical sciences and was considered exempted.

## Results

Thirty-five residents participated in the study, 27 male and 8 females. Their average age was 29 years (ranging from 25 to 34). Nineteen of them were second year residents and 16 were third year.

The average score of residents' performance before the workshop was 5.21 (out of 100), which increased significantly to 66.1 after the workshop (*p* value < 0.01). Also, the average knowledge of residents, which was measured as self-reported, increased significantly from 28.85 to 71.09 (out of 100) (*p* value < 0.01).

In the third part of the questionnaire, which examined students' attitudes and interests, the average score significantly increased from 40.49 to 74.81 (out of 100) (*p* value:0.03).

## Discussion

Lecture-based teaching is the most common method for presenting up-to-date information in learning process, but it has been perceived to have low educational yield, as it fast-forwards the student from problem to solution and focuses on memorizing, ignoring the analytical way of thinking, so it promotes shallow learning. Alternative active learning models have been introduced to address these concerns. Some of these methods to stimulate student’s engagement in learning process are video-based learning, group working encouragement, problem-based learnings, flipped classrooms, workshops and laboratories^2–5^.

There are advocates for active learning in clinical optics, but few studies have been conducted in this regard. Tu et al. investigated the effectiveness of flipped classroom versus lecture-based clinical optics curricula in an ophthalmology resident’s education setting. They showed that flipped classroom approach to clinical optics education was associated with higher performance on the optics subsection of the Ophthalmic Knowledge Assessment Program (OKAP) examination, and they suggested that flipped classroom approach may be more effective than traditional lectures for teaching clinical optics^6^. Some innovative workshops and experienced-based approaches have been proposed in optometry curricula. Putnam has demonstrated laboratory activities and problem solving sessions in optics curriculum at the Arizona College of Optometry that aims to provide students with an understanding of geometrical, physical, and visual optics principals^7^. Faheemah Saeed described hands-on learning of geometric optical principles by small groups of students within the large lecture setting. She used table-top equipment (lenses, mirrors, prisms and laser sources) to increase visualization of optical principles and students’ engagement in the learning process and reported that it was well-received in the majority of students. However, in this paper, only the geometrical optics was practiced and the improvement of students' performance by changing the teaching method was not studied objectively^8^.

As we believe extending the concepts learned in the classroom via lectures to the lab can facilitate the learning process of students, this lab was set up for this purpose. Its positive results have been observed from the first year. In the early years, students were evaluated practically in the lab in each session and with a written exam at the end of the course. During those years, evaluation of students' scores and feedback from students and professors showed the better effect of this type of education than lectures. From 2017, we decided to do a more objective assessment of students, so pre-posttest with a valid and reliable questionnaire was designed. The questionnaire consisted of 3 parts: questions that assessed self-estimation of the residents from their optical knowledge, open-style questions to evaluate their knowledge and performance objectively and questions to estimate the attitude of residents towards gaining practical knowledge in the field of optics. We saw significant improvements in all 3 parts in this study. Also, in a poll at the end of the course, most of the students reported satisfaction with the workshop and tests provided in the lab in terms of usefulness of the materials and appropriateness of information. They also reported increased awareness of the importance of the optics knowledge in their practice and their willingness to implement this knowledge in their regular practice.

The strengths of this study are: (1) Contrary to the previous studies that we mentioned, in this study and in our lab, we tried to cover most of important optical issues in the field of work of an ophthalmologist with standard tests, and we did not just teach geometrical optics. (2) Students performed all the experiments in small groups in a well-equipped laboratory. (3) An ophthalmology professor and an optics professor were both present in the lab, to demonstrate how optical concepts and their clinical applications are connected. (4) Knowledge, performance and attitude of students were evaluated with a pre-posttest study.

The limitations of our study are that our data are from a single institution, and we did not have a control group to compare our results with other teaching methods such as lecturing. However, because these students were in their second and third year of residency, they all had attended optics classes the year before, and considering the short duration of this workshop, their change in performance in this study was measured with a pre-posttest. In this study, we did not create a control group because we knew from our experiences in previous years that participating in this course would be beneficial for residents and depriving a group of students was not ethical. Therefore, we were satisfied with a before-after study.

## Conclusion

The present study has addressed that training workshops and labs are effective to bring about change in knowledge and attitude of ophthalmology residents toward optics as a new teaching strategy that could be implemented in their curriculum. This was consistent with previous literatures that have reported workshops were helpful to enhance knowledge, practice and the attitude of students. So it seems that ophthalmology education can be improved by utilization of new teaching strategies.

## Data Availability

The datasets used and/or analysed during the current study are available from the corresponding author on reasonable request.
